# eGFR partially mediates the association between Dietary Inflammatory Index and all-cause mortality in older adults: A retrospective cohort study based on NHANES 2001–2018

**DOI:** 10.1097/MD.0000000000050390

**Published:** 2026-08-28

**Authors:** Tingting Hu, Ying Zhang, Zhu Chen, Zhifen Zhang, Jun Su

**Affiliations:** a Department of Clinical Laboratory, Hangzhou Women’s Hospital (Hangzhou Maternity and Child Health Care Hospital), Hangzhou, Zhejiang, China; b Department of Psychosomatic Diseases (II), Affiliated Mental Health Center & Hangzhou Seventh People’s Hospital, Zhejiang University School of Medicine, Hangzhou, Zhejiang, China; c Department of Imaging Sciences, Zhejiang Greentown Cardiovascular Hospital, Hangzhou, Zhejiang, China; d Department of Gynaecological Endocrinology Centre, Hangzhou Women’s Hospital (Hangzhou Maternity and Child Health Care Hospital), Hangzhou, Zhejiang, China; e Department of Intensive Care, Hangzhou Women’s Hospital (Hangzhou Maternity and Child Health Care Hospital), Hangzhou, Zhejiang, China.

**Keywords:** all-cause mortality, Dietary Inflammatory Index, elderly population, estimated glomerular filtration rate, mediation analysis

## Abstract

This study examines the association between the Dietary Inflammatory Index (DII) and all-cause mortality in older adults and investigates the mediating role of estimated glomerular filtration rate (eGFR) using National Health and Nutrition Examination Survey data. We included 6300 participants aged ≥ 60 years from 9 National Health and Nutrition Examination Survey cycles (2001–2018). All-cause mortality (ascertained via linkage to the National Center for Health Statistics) was the primary endpoint. Cox proportional hazards models evaluated the association. Mediation analysis (mediation package, R 4.4.0) quantified eGFR’s contribution. The average DII of the enrolled subjects was 0.73 (0.04), and the average age was 68.84 (0.13) years. Over 116.4 months of follow-up, 1746 all-cause deaths occurred. Each 1-unit DII increase increased all-cause mortality risk by 5.6% (adjusted hazard ratio = 1.056, 95% confidence interval [CI]:1.020–1.094; *P* = .002). The highest DII tertile had 26% higher all-cause mortality vs the lowest (hazard ratio = 1.26, 95% CI:1.08–1.48). eGFR mediated 9.67% (95% CI:3.65–25.7%) of the DII-all-cause mortality association. Pro-inflammatory diets independently increase all-cause mortality in older adults, partially mediated through eGFR. Dietary interventions and renal monitoring may reduce this risk.

## 1. Introduction

The accelerating global aging process has made health issues among individuals aged 60 and above a public health focal point. This population faces the dual challenges of high prevalence of chronic diseases and increased all-cause mortality. Diet, as a modifiable lifestyle factor, plays a crucial role in delaying aging and improving prognosis.^[1]^ Evidence confirms that healthy dietary patterns, particularly those consistent with World Health Organization guidelines, correlate with lower all-cause mortality in older European and North American populations.^[2]^ Furthermore, healthy diets mitigate risks of multiple chronic diseases, thereby extending health span, which is defined as the period free from major illness.^[3]^

Chronic low-grade inflammation is a salient feature of the aging process and is closely linked to a variety of age-related diseases, such as cardiovascular disease, cancer, and renal dysfunction.^[4]^ This chronic inflammatory state may be driven by a variety of factors, including pro-inflammatory diets, increased oxidative stress, and the decline of immune system function.^[5,6]^ Among them, pro-inflammatory diets induce oxidative stress and mitochondrial damage, which in turn activate the nuclear factor-kappa B pathway, promoting the release of pro-inflammatory cytokines such as tumor necrosis factor-alpha (TNF-α), IL-1β, and interleukin-6 (IL-6), leading to a chronic low-grade inflammatory state and ultimately resulting in tissue damage and aging-related diseases.^[5,7]^ The Dietary Inflammatory Index (DII) is a tool for assessing the inflammatory potential of diet, which quantifies the impact of dietary components on inflammatory biomarkers. The DII has been used to study the relationship between diet and various chronic diseases, including cardiovascular disease, cancer, and chronic kidney disease (CKD), providing a standardized tool for evaluating the inflammatory potential of diet.^[8,9]^ Studies have shown that a higher DII is positively correlated with all-cause mortality,^[10]^ but the heterogeneity of this conclusion in the elderly population is worth paying attention to Metabolic changes during aging, immune senescence, and multimorbidity may significantly affect the association between diet, inflammation, and health outcomes.

Diet-induced chronic low-grade inflammation can cause damage to multiple organ systems.^[11]^ The kidneys, as high-perfusion organs, play a crucial role in the excretion of metabolic waste and the regulation of homeostasis. Their high vulnerability makes them particularly susceptible to attacks from inflammatory mediators and oxidative stress, which in turn induce glomerulosclerosis and renal tubular interstitial fibrosis, eventually leading to progressive renal function decline.^[12]^ The estimated glomerular filtration rate (eGFR) is the most widely used quantitative indicator of renal function in clinical practice and epidemiological studies, and its decline is a sensitive marker of renal function impairment.^[13]^ Existing evidence clearly shows a significant correlation between a higher DII and lower eGFR levels.^[14]^ Notably, reduced eGFR has been established as a powerful independent predictor of all-cause mortality, with this association being particularly pronounced in the elderly population, who experience age-related physiological decline in renal function.^[15]^ Given that DII may increase mortality risk by impairing renal function (manifested as a decline in eGFR), assessing the potential mediating role of eGFR in the association between DII and all-cause mortality is crucial for a deeper understanding of its pathogenic mechanisms. Therefore, this study aims to fill this key gap using National Health and Nutrition Examination Survey (NHANES) data and to test the hypothesis that eGFR partially mediates the effect of DII on all-cause mortality.

## 2. Materials and methods

### 2.1. Study population

**Figure 1.**
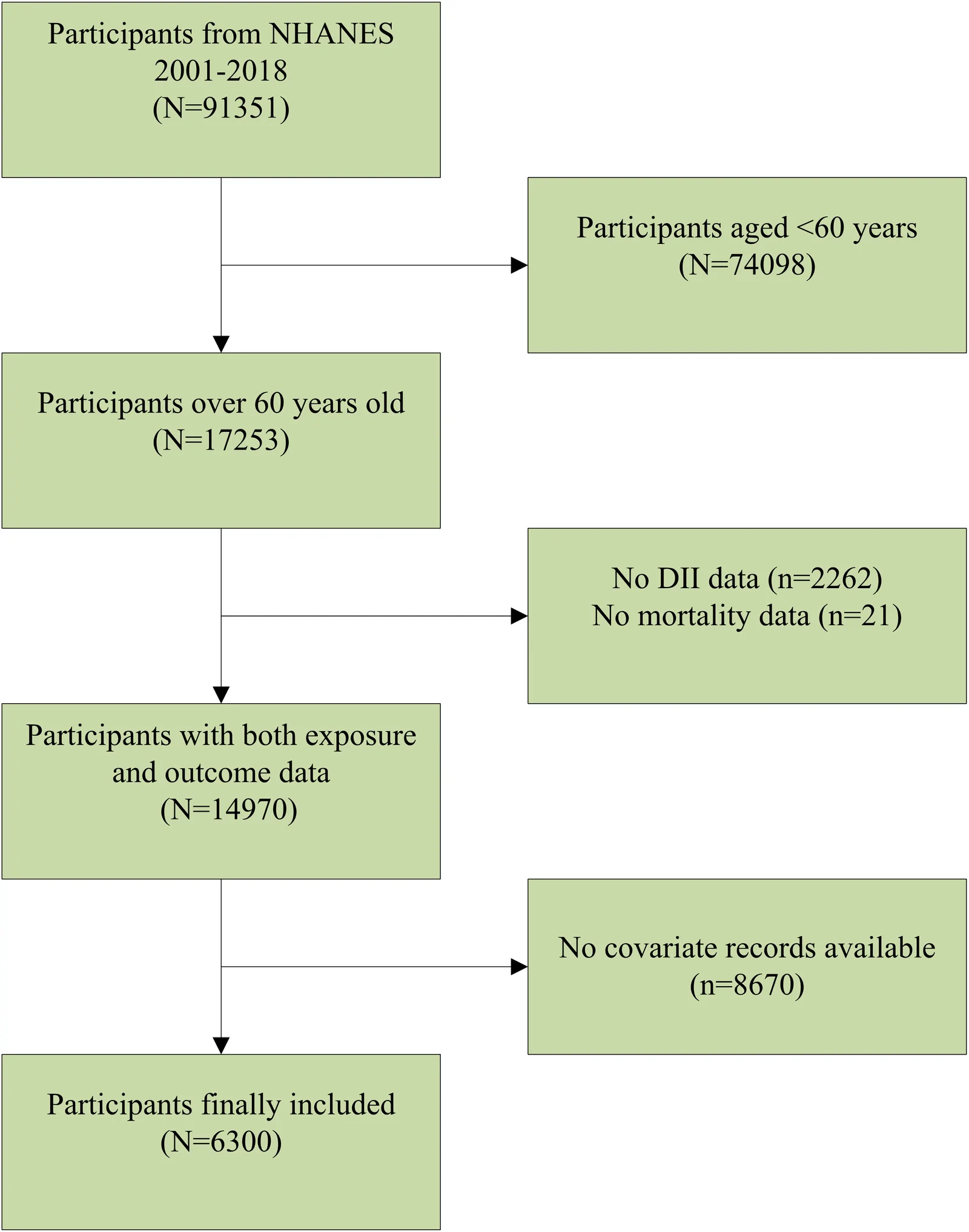
Flowchart of study participants. DII = Dietary Inflammatory Index, NHANES = National Health and Nutrition Examination Survey.

The NHANES is a population-based national survey that provides information on the nutrition and health of the U.S. population. The NHANES database is publicly available at www.cdc.gov/nchs/nhanes. Survival status of NHANES participants is collected in the National Death Index. Our study combined data from NHANES across 9 cycles from 2001 to 2018. Among 91,351 participants, 74,098 were under the age of 60, 2262 had missing data on the DII, 21 had missing mortality outcome data, and 8670 had missing covariate data. After applying these exclusion criteria, 6300 participants were included in the clinical analysis (Fig. 1).

### 2.2. Assessment of DII

NHANES collects dietary data through a standardized procedure, specifically using data from two 24-hour dietary recall interviews – the first conducted in-person at the Mobile Examination Center and the second by telephone.^[16]^ In this study, we quantitatively analyzed participants’ nutrient intake (excluding the influence of supplements and medications). If both data points were valid, the mean value was used; if only 1 data point was valid, the single data point was adopted. The DII was calculated based on the assessment system proposed by Shivappa et al.^[17]^ The original model of this system includes 45 dietary parameters. As noted in a previous NHANES-based study,^[18]^ even if the number of nutrients used to calculate the DII is <30, the DII score remains valid and available. Limited by the coverage of dietary components in the NHANES database, this study ultimately selected 27 key indicators to construct the DII model, which specifically include: macronutrients (carbohydrates, protein, total fat), fatty acid spectrum (saturated, monounsaturated, polyunsaturated fatty acids and their subclasses), vitamin groups (A, C, D, E, and B vitamins), minerals (iron, magnesium, zinc, and selenium), dietary fiber, cholesterol, caffeine, and alcohol intake. Among them, the n-3 fatty acid components include alpha-linolenic acid (18:3), stearidonic acid (18:4), EPA (20:5), DPA (22:5), and DHA (22:6); the n-6 fatty acid components are composed of linoleic acid (18:2) and arachidonic acid (20:4).^[19]^ The DII scoring method follows the standardized algorithm of reference,^[17]^ and the quantification is achieved by comparing dietary parameters with the global dietary inflammatory benchmark database.

The DII was calculated using the formula:


DII=(Z score×the inflammatory effectscore of each dietary component)

$$\begin{array}{c} \begin{array}{cc} Z\;score=\left(daily\;mean\;intake-global\;\;daily\;mean\;intake\right)/standard\;deviation\\ & Z\;score=Z\;score\to \left(converted\;to\;\;a\;percentile\;score\right)\times 2-1 \end{array} \end{array}$$

### 2.3. Assessment of all-cause mortality

In this study, the primary outcome was all-cause mortality. To determine mortality status, the NHANES public-use linked mortality file through December 31, 2019, was used in conjunction with the National Death Index via a probabilistic matching algorithm implemented by the National Center for Health Statistics (NCHS).

### 2.4. Covariates

In this study, the following covariates were collected. Continuous variables included age, family poverty-to-income ratio (PIR), body mass index (BMI), alanine aminotransferase (ALT), aspartate aminotransferase (AST), fasting blood glucose (FBG), hemoglobin A1c (HbA1c), uric acid (UA), serum creatinine (SCr), total cholesterol (TC), vitamin D, and eGFR. Categorical variables included gender, race, education level, smoking status, drinking status, hypertension status (yes/no), and diabetes mellitus (DM) status (yes/no). Among these, the calculation method for eGFR is as follows:


$$\begin{array}{c} \begin{array}{cc} eGFR=141\times min{\left(Scr/\kappa ,1\right)}^{\alpha }\times max{\left(Scr/\kappa ,1\right)}^{-1.209}\\ & \times 0.993^{Age}\times \left[1.018\left(if\;Female\right)\right]\times \left[1.159\left(if\;Black\right)\right], \end{array} \end{array}$$

where Scr is serum creatinine (mg/dL); for females: *k* = 0.7, α = −0.329; for males: *k* = 0.9, α = −0.411; min denotes the minimum value of Scr/*k* or 1; max denotes the maximum value of Scr/*k* or 1.^[20]^ Hypertension was defined as having a history of hypertension or a systolic blood pressure> 140 mm Hg or a diastolic blood pressure > 90 mm Hg.^[21]^ DM was defined as having a history of diabetes, using insulin, taking diabetes medications to lower blood glucose, a HbA1c ≥ 6.5, a FBG ≥ 126 mg/dL, or a 2-h postprandial blood glucose ≥ 200 mg/dL.^[22]^ Smoking status was classified into 3 categories based on previous studies: never smokers (individuals who had smoked fewer than 100 cigarettes in their lifetime), former smokers (individuals who had smoked more than 100 cigarettes in their lifetime but had quit smoking), and current smokers (individuals who were still smoking at the time of the study).^[23]^ Drinking status was based on the average daily alcohol intake in the past 12 months, with different thresholds set for males and females,^[24]^ mild drinking was defined as ≤ 1 drink per day for females and ≤ 2 drinks per day for males; moderate drinking was defined as 1 to 3 drinks per day for females and 2 to 4 drinks per day for males; heavy drinking was defined as ≥ 4 drinks per day for females and ≥ 5 drinks per day for males. Education level was divided into 3 groups based on the NHANES questionnaire data: less than high school, high school graduate or equivalent, and some college or above.^[25]^

### 2.5. Statistical analysis

To enhance the representativeness of the study findings, we adhered to the NHANES-recommended weighting procedures in the data analysis. In accordance with the complex, multistage probability sampling design of NHANES, all analyses incorporated strata, primary sampling units, and appropriate sample weights to generate nationally representative estimates and ensure generalizability to the noninstitutionalized U.S. population. Analyses were performed using the “survey” package in R. Weight selection followed NHANES guidelines, prioritizing variables representative of relevant population subgroups. For indicators derived from the Mobile Examination Center, such as FBG, the corresponding subsample weight (WTSAF2YR) was applied. For dietary intake data derived from 24-hour recalls, the day 1 dietary weights (WTDR2D) were applied. Data processing and analysis were performed using R version 4.4.0 (April 24, 2024), along with Storm Statistical Platform (www.medsta.cn/software). The normality of continuous variables was assessed using the Kolmogorov-Smirnov test and visual inspection of histograms/Q-Q plots. Normally distributed variables were presented as mean (SE) and compared using analysis of variance, while non-normally distributed variables were presented as median (interquartile range) and compared using the Kruskal–Wallis test. Categorical data were expressed as n (%) and analyzed using chi-square tests or Fisher exact test. A *P*-value of <.05 was considered statistically significant. Univariate and multivariate Cox proportional hazards models were used to evaluate the association between the DII and all-cause mortality, with results presented as hazard ratios (HR) and 95% confidence intervals (CI). Model 1 was unadjusted. Model 2 was adjusted for gender, age, and race. Based on Model 2, Model 3 further adjusted for smoking status, education level, drinking status, hypertension status, diabetes status, BMI, ALT, AST, FBG, HbA1c, UA, SCr, TC, and Vitamin D. Kaplan–Meier curves were used to estimate survival over time for the 3 DII categories, and the log-rank test was used to assess differences between the survival curves. Additionally, Cox proportional hazards regression models were used for subgroup analyses to evaluate the heterogeneity of the association between DII and all-cause mortality across different subgroups. Subgroup analyses were based on the following stratification variables: age, gender, race, BMI, education level, smoking status, drinking status, hypertension status, and diabetes status. Interaction terms were tested to assess whether these subgroup variables significantly modified the association between DII and mortality. The *P*-value for interaction was calculated using the likelihood ratio test, with results presented as HR and 95% CI. A *P*-value for interaction <.05 was considered to indicate a significant modifying effect of the subgroup variable on the association between DII and mortality.

Mediation analysis was performed to examine whether eGFR mediated the association between DII and all-cause mortality. Three regression models were constructed: a total effect model (DII → mortality, without eGFR), a mediator model (DII → eGFR using linear regression), and a direct effect model (DII + eGFR → mortality). The indirect effect was estimated using the bootstrap method with 1000 resamples, and its significance was determined by whether the 95% CI excluded zero. All models were adjusted for gender, age, race, education level, smoking, drinking, hypertension, DM, PIR, BMI, ALT, AST, FBG, HbA1c, TC, and vitamin D.

### 2.6. Ethics statement

This study utilized publicly available, de-identified NHANES data provided by the NCHS, Centers for Disease Control and Prevention. All procedures of the NHANES project were approved by the NCHS Research Ethics Review Board and informed consent was obtained from all participants. The data use and analysis in this study strictly adhered to the NHANES data use agreement. The NHANES ethical approval and informed consent can be found at the following links: https://www.cdc.gov/nchs/nhanes/about/erb.html and https://www.cdc.gov/nchs/nhanes/irba98.htm. Therefore, this study was exempt from further approval by an institutional ethics review board.

## 3. Results

### 3.1. Participants characteristics

After applying the inclusion and exclusion criteria, a total of 6300 participants were eligible for the study. The mean DII value was 0.73 (0.04). The weighted demographic characteristics of participants across the DII tertiles (T1: DII < −0.08, T2: −0.08 ≤ DII < 1.80, T3: DII ≥ 1.80) are presented in Table 1. Significant differences were observed in gender, race, PIR, BMI, HbA1c, UA, TC, vitamin D, eGFR, smoking status, education level, drinking status, hypertension status, diabetes status, and all-cause mortality across the different DII groups (tertiles, T1–T3). Compared with those in the lowest tertile, participants in the highest tertile were more likely to be female, have a higher BMI, have an education level below college, be current smokers, have hypertension, and have diabetes, with higher levels of HbA1c, UA, and TC. Conversely, they exhibited lower PIR and eGFR levels (Table 1). Additionally, the all-cause mortality rate was higher in the T3 group.

**Table 1:** Characteristics of the study population with various DII tertiles.

Characteristics	Total	Tertiles of DII			*P* value
	(n = 6300)	T1 (n = 1926)	T2 (n = 2140)	T3 (n = 2234)	
Mean (SE)					
PIR	3.41 (0.04)	3.75 (0.06)	3.42 (0.05)	3.06 (0.05)	<.001
BMI, kg/m^2^	28.91 (0.12)	28.23 (0.18)	29.08 (0.21)	29.41 (0.17)	<.001
ALT, U/L	22.90 (0.22)	23.38 (0.44)	22.88 (0.33)	22.44 (0.42)	.130
AST, U/L	25.06 (0.19)	25.07 (0.30)	24.99 (0.30)	25.11 (0.36)	.923
UA, umol/L	338.23 (1.40)	333.02 (2.62)	338.93 (2.77)	342.69 (2.40)	.009
SCr, umol/L	85.20 (0.56)	84.04 (0.88)	85.75 (0.94)	85.80 (0.86)	.120
TC, mmol/L	5.17 (0.02)	5.06 (0.04)	5.19 (0.04)	5.25 (0.03)	<.001
Vitamin D, nmol/L	76.03 (0.66)	78.85 (1.27)	76.30 (0.94)	72.94 (1.05)	<.001
eGFR, mL/min/1.73m^2^	79.47 (0.39)	82.72 (0.69)	78.87 (0.54)	76.83 (0.66)	<.001
M (Q_1_, Q_3_)					
Age, yrs	68.00 (63.00–74.00)	67.00 (63.00–73.00)	68.00 (63.00–74.00)	68.00 (63.00–74.00)	.164
FBG, mmol/L	5.33 (4.94–6.00)	5.33 (4.88–5.94)	5.38 (5.00–6.00)	5.33 (4.88–6.05)	.268
HbA1c, %	5.60 (5.40–5.90)	5.60 (5.30–5.90)	5.60 (5.40–6.00)	5.60 (5.40–6.00)	<.001
n (%)					
Gender					<.001
Male	3584 (50.29)	1238 (59.85)	1236 (50.05)	1110 (41.00)	
Female	2716 (49.71)	688 (40.15)	904 (49.95)	1124 (59.00)	
Race					<.001
Mexican American	785 (3.22)	239 (2.96)	262 (3.30)	284 (3.39)	
Other Hispanic	463 (2.62)	118 (2.08)	168 (2.72)	177 (3.06)	
Non-Hispanic White	3711 (85.21)	1199 (87.19)	1285 (85.47)	1227 (82.96)	
Non-Hispanic Black	1037 (5.87)	253 (4.28)	334 (5.42)	450 (7.93)	
Other Race	304 (3.08)	117 (3.49)	91 (3.10)	96 (2.66)	
Smoking status					<.001
Never	2575 (43.22)	808 (45.60)	881 (44.06)	886 (39.97)	
Former	2802 (44.46)	923 (46.83)	964 (44.44)	915 (42.11)	
Now	923 (12.33)	195 (7.57)	295 (11.49)	433 (17.93)	
Education level					<.001
Less than high school	1424 (13.06)	318 (8.40)	469 (12.77)	637 (18.00)	
High school or equivalent	1495 (23.63)	365 (17.26)	510 (23.42)	620 (30.19)	
College or above	3381 (63.32)	1243 (74.34)	1161 (63.81)	977 (51.81)	
Drinking status					.008
Mild drinking	4428 (71.96)	1420 (74.85)	1491 (72.15)	1517 (68.87)	
Moderate drinking	1440 (23.47)	413 (22.04)	492 (22.90)	535 (25.48)	
Heavy drinking	432 (4.57)	93 (3.10)	157 (4.96)	182 (5.65)	
Hypertension					.007
No	1987 (34.77)	649 (38.54)	684 (33.65)	654 (32.17)	
Yes	4313 (65.23)	1277 (61.46)	1456 (66.35)	1580 (67.83)	
DM					.048
No	4554 (77.36)	1421 (79.67)	1546 (77.17)	1587 (75.25)	
Yes	1746 (22.64)	505 (20.33)	594 (22.83)	647 (24.75)	
All-cause mortality					<.001
No	4554 (76.59)	1486 (81.65)	1557 (76.61)	1511 (71.52)	
Yes	1746 (23.41)	440 (18.35)	583 (23.39)	723 (28.48)	

ALT = alanine aminotransferase, AST = aspartate aminotransferase, BMI = body mass index, DII = Dietary Inflammatory Index, DM = diabetes mellitus, eGFR = estimated glomerular filtration rate, FBG = fasting blood glucose, HbA1c = hemoglobin A1c, PIR = ratio of family income to poverty, SCr = serum creatinine, T = tertiles, TC = total cholesterol, UA = uric acid, Vitamin D = 25OHD2 + 25OHD3.

### 3.2. Associations of DII with all-cause mortality

**Figure 2.**
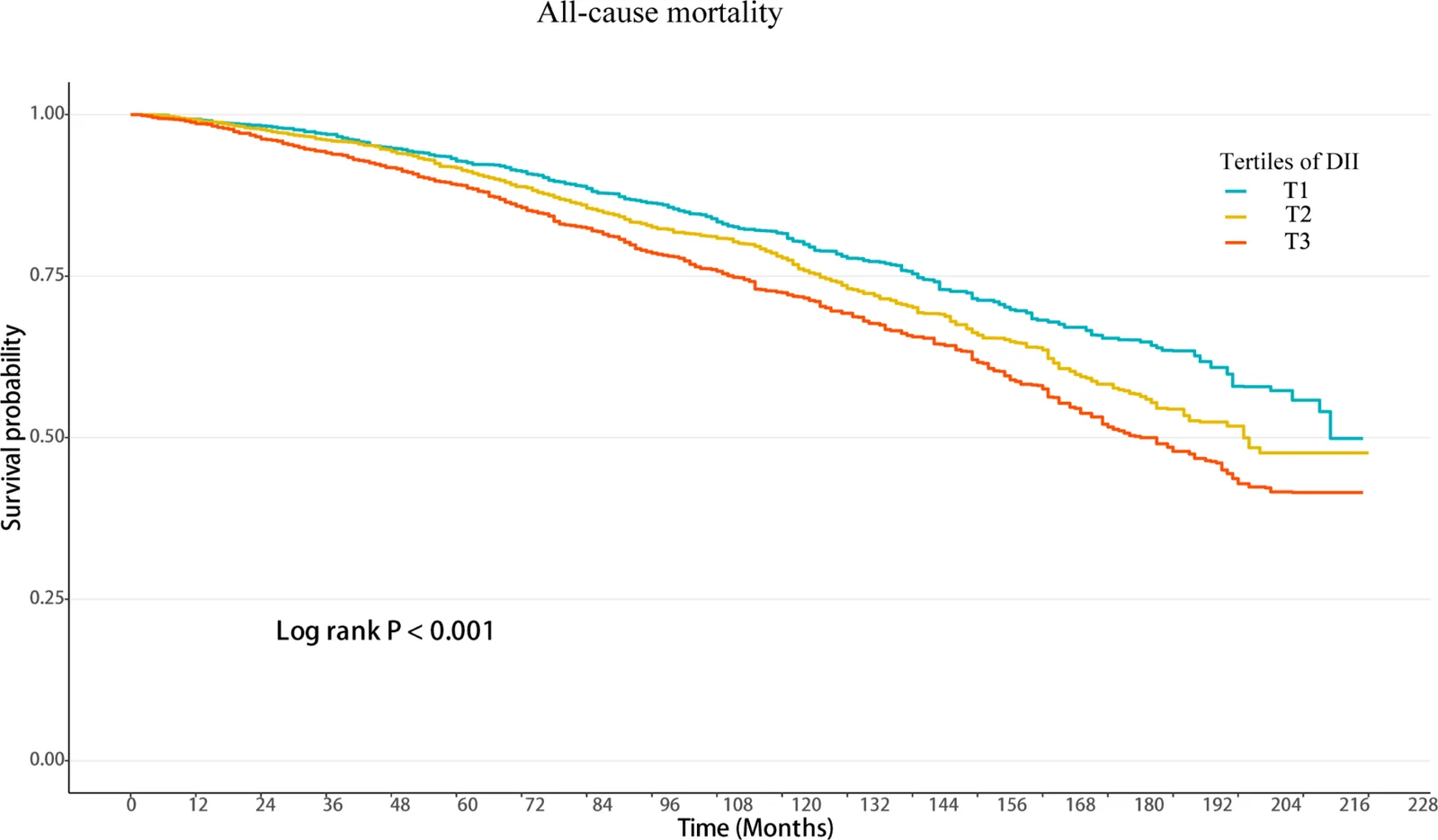
Kaplan–Meier curves of the survival rate of participants with DII tertiles. DII = Dietary Inflammatory Index.

Table 2 shows the association between the DII and all-cause mortality. In Model 1, DII was significantly associated with all-cause mortality (HR = 1.100, 95% CI:1.057–1.144). In Model 2 (adjusted for age, gender, and race) and Model 3 (further adjusted for multiple covariates), the association remained significant (HR = 1.114, 95% CI:1.076–1.153 and HR = 1.056, 95% CI:1.020–1.094, respectively). The *P*-value for trend was <.05 in all models (all *P* < .05), indicating a dose–response relationship between DII and all-cause mortality. The Kaplan–Meier survival curves in Figure 2 also showed that participants in the higher DII tertiles had lower survival rates, with significant differences between groups according to the log-rank test (*P* < .001). In the fully adjusted Model 3, the HRs for T2 and T3 were 1.126 (95% CI:0.966–1.312) and 1.258 (95% CI:1.072–1.477), respectively, with the association in T3 remaining statistically significant.

**Table 2:** Cox regression analysis of the relationship between DII and all-cause mortality.

Variables	Model 1 [HR (95% CI)]	Model 2 [HR (95% CI)]	Model 3 [HR (95% CI)]
DII (continuous)	1.100 (1.057–1.144)	1.114 (1.076–1.153)	1.056 (1.020–1.094)
*P* for trend	<.001	<.001	.002
DII (Tertile)			
Tertile 1	1.000 (Reference)	1.000 (Reference)	1.000 (Reference)
Tertile 2	1.245 (1.055–1.468)	1.231 (1.051–1.443)	1.126 (0.966–1.312)
Tertile 3	1.531 (1.270–1.846)	1.596 (1.357–1.876)	1.258 (1.072–1.477)
*P* for trend	<.001	<.001	.005

Model 1: no covariates were adjusted. Model 2: age, gender, and Race were adjusted. Model 3: age, gender, Race, PIR, BMI, education level, smoking status, drinking status, hypertension, DM, ALT, AST, FBG, HbA1c, UA, Scr, TC, and Vitamin D were adjusted.

ALT = alanine aminotransferase, AST = aspartate aminotransferase, BMI = body mass index, CI = confidence interval, DII = Dietary Inflammatory Index, DM = diabetes mellitus, FBG = fasting blood glucose, HbA1c = hemoglobin A1c, HR = hazard ratio, PIR = ratio of family income to poverty, SCr = serum creatinine, TC = total cholesterol, UA = uric acid, Vitamin D = 25OHD2 + 25OHD3.

### 3.3. Subgroup analysis

The study conducted subgroup analyses to evaluate the stability of the association between DII and all-cause mortality across different population strata (see Table 3). The results indicated that in the age subgroups, DII was significantly positively correlated with all-cause mortality across different age categories. In the gender subgroups, both males and females exhibited a significant positive correlation. In the BMI subgroups, a significant positive correlation was observed among individuals with normal weight and those who were overweight, while the association was not significant among obese individuals. Additionally, in the race subgroups, Non-Hispanic Whites and Non-Hispanic Blacks showed a general positive correlation trend between DII and all-cause mortality. The same trend was observed among individuals with a college education or above in the education level subgroup, among never smokers and former smokers in the smoking status subgroup, and among mild drinkers and heavy drinkers in the drinking status subgroup. The association was also generally positive among individuals with or without hypertension or diabetes. However, the interactions between subgroups were not significant, suggesting that these factors may not have a significant interactive impact on the association between DII and all-cause mortality.

**Table 3:** Subgroup analysis of associations between DII and all-cause mortality.

Variables	n (%)	HR (95% CI)	*P*	*P* for interaction
Age (yrs)				.239
60 ≤ Age < 70	3428 (54.41)	1.14 (1.05–1.24)	.002	
70 ≤ Age < 80	1919 (30.46)	1.07 (1.01–1.14)	.02	
>80	953 (15.13)	1.05 (1.01–1.10)	.013	
Gender				.217
Male	3584 (56.89)	1.10 (1.05–1.15)	<.001	
Female	2716 (43.11)	1.15 (1.08–1.23)	<.001	
Race				.863
Mexican American	785 (12.46)	1.12 (0.98–1.28)	.09	
Other Hispanic	463 (7.35)	1.04 (0.82–1.33)	.736	
Non-Hispanic White	3711 (58.90)	1.10 (1.05–1.15)	<.001	
Non-Hispanic Black	1037 (16.46)	1.16 (1.06–1.26)	.001	
Other Race	304 (4.83)	1.12 (0.91–1.37)	.275	
BMI (kg/m^2^)				.057
Normal, <25	1629 (25.86)	1.14 (1.06–1.22)	<.001	
Overweight, 25–30	2444 (38.79)	1.14 (1.08–1.21)	<.001	
Obese, >30	2227 (35.35)	1.03 (0.96–1.10)	.417	
PIR				.922
<1.3	1292 (20.51)	1.06 (0.97–1.15)	.187	
1.3–3.5	2602 (41.30)	1.08 (1.01–1.15)	.03	
>3.5	2406 (38.19)	1.06 (1.01–1.12)	.021	
Education level				.965
Less than high school	1424 (22.60)	1.08 (0.99–1.17)	.079	
High school or equivalent	1495 (23.73)	1.06 (0.99–1.15)	.107	
College or above	3381 (53.67)	1.08 (1.02–1.14)	<.001	
Smoking				.114
Never	2575 (40.87)	1.10 (1.03–1.18)	.005	
Former	2802 (44.48)	1.12 (1.07–1.17)	<.001	
Now	923 (14.65)	0.99 (0.89–1.11)	.887	
Drinking				.703
Mild drinking	4428 (70.29)	1.10 (1.05–1.16)	<.001	
Moderate drinking	1440 (22.86)	1.08 (0.99–1.19)	.093	
Heavy drinking	432 (6.86)	1.17 (1.03–1.32)	.013	
Hypertension				.571
No	1987 (31.54)	1.11 (1.02–1.21)	.013	
Yes	4313 (68.46)	1.08 (1.04–1.13)	<.001	
DM				.96
No	4554 (72.29)	1.09 (1.04–1.14)	<.001	
Yes	1746 (27.71)	1.10 (1.04–1.17)	.002	

BMI = body mass index, CI = confidence interval, DII = Dietary Inflammatory Index, DM = diabetes mellitus, HR = hazard ratio, PIR = ratio of family income to poverty.

### 3.4. Associations of eGFR with DII and all-cause mortality

Table 4 shows the association between DII and eGFR after multivariable linear regression. After adjusting for all variables, DII was negatively correlated with eGFR (β = −0.009, 95% CI [−0.013 to −0.006], *P* < .001). Table 5 presents the results of the Cox regression analysis of the association between eGFR and all-cause mortality. After adjusting for all variables, eGFR remained negatively correlated with all-cause mortality (HR = 0.993, 95% CI [0.989–0.997], *P* < .001).

**Table 4:** The associations between DII and eGFR.

	*β* value	95% CI	*P* value
eGFR			
Model 1	−0.013	−0.017 to −0.010	<.001
Model 2	−0.009	−0.013 to −0.005	<.001
Model 3	−0.009	−0.013 to −0.006	<.001

Model 1: no covariates were adjusted. Model 2: age, gender, and Race were adjusted. Model 3: age, gender, Race, PIR, BMI, education level, smoking status, drinking status, hypertension, DM, ALT, AST, FBG, HbA1c, TC, and Vitamin D were adjusted.

ALT = alanine aminotransferase, AST = aspartate aminotransferase, BMI = body mass index, CI = confidence interval, DII = Dietary Inflammatory Index, DM = diabetes mellitus, eGFR = estimated glomerular filtration rate, FBG = fasting blood glucose, HbA1c = hemoglobin A1c, PIR = ratio of family income to poverty, TC = total cholesterol, Vitamin D = 25OHD2 + 25OHD3.

**Table 5:** The associations of eGFR with all-cause mortality.

	Model 1	*P*	Model 2	*P*	Model 3	*P*
	HR (95% CI)		HR (95% CI)		HR (95% CI)	
All-cause mortality						
eGFR	0.982 (0.979–0.986)	<.001	0.992 (0.988–0.996)	<.001	0.993 (0.989–0.997)	<.001

Model 1: no covariates were adjusted. Model 2: age, gender, and Race were adjusted. Model 3: age, gender, Race, PIR, BMI, education level, smoking status, drinking status, hypertension, DM, ALT, AST, FBG, HbA1c, TC, and Vitamin D were adjusted.

ALT = alanine aminotransferase, AST = aspartate aminotransferase, BMI = body mass index, CI = confidence interval, DM = diabetes mellitus, eGFR = estimated glomerular filtration rate, FBG = fasting blood glucose; HbA1c = hemoglobin A1c, HR = hazard ratio, PIR = ratio of family income to poverty, TC = total cholesterol, Vitamin D = 25OHD2 + 25OHD3.

### 3.5. Mediation analysis

**Figure 3.**
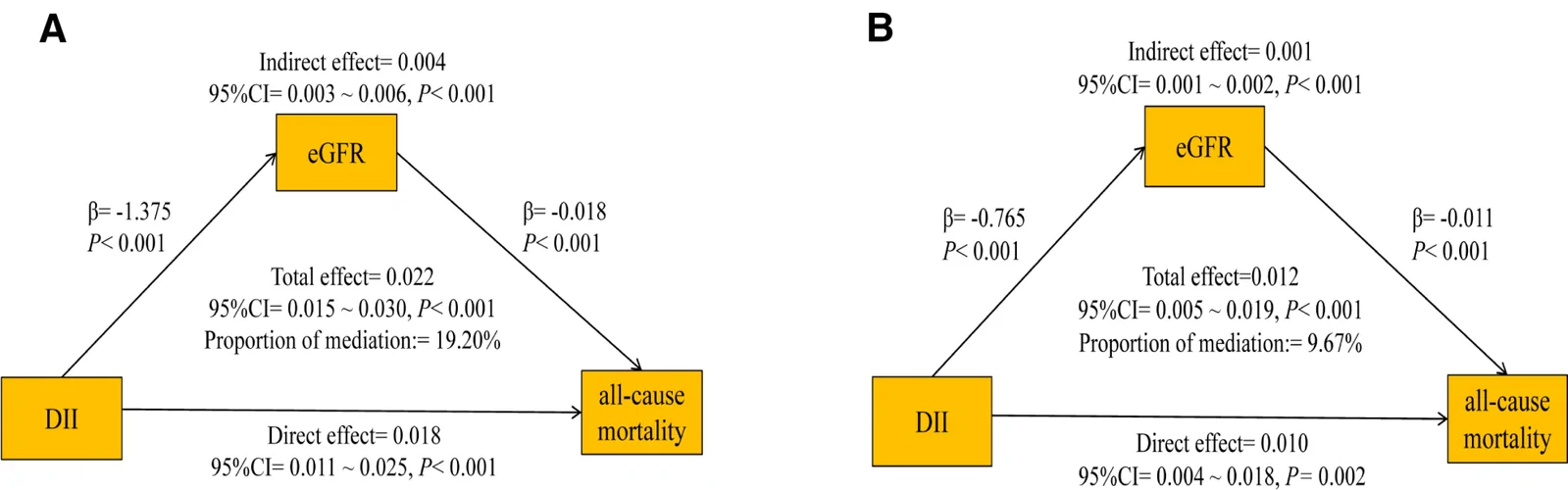
Analysis of the mediation by eGFR of the associations of DII with all-cause mortality. (A) Unadjusted model and (B) adjusted for gender, age, race, education level, smoking, drinking, hypertension, diabetes mellitus, PIR, BMI, ALT, AST, FBG, HbA1c, TC, and vitamin D. ALT = alanine aminotransferase, AST = aspartate aminotransferase, BMI = body mass index, CI = confidence interval, DII = dietary Inflammatory Index, eGFR = estimated glomerular filtration rate, FBG = fasting blood glucose, HbA1c = hemoglobin A1c, PIR = ratio of family income to poverty, TC = total cholesterol.

The mediation analysis results are shown in Figure 3. The total effect of DII on all-cause mortality was 0.012 (95% CI: 0.005 to 0.019, *P* < .001). In the mediator model, DII was significantly associated with eGFR (*β* = −0.765, 95% CI: −1.073 to −0.457, *P* < .001). In the direct effect model, after adjusting for eGFR, the direct effect of DII on mortality was 0.010 (95% CI: 0.004 to 0.018, *P* = .002), and eGFR remained significantly associated with all-cause mortality (*β* = −0.011, 95% CI: −0.016 to −0.006, *P* < .001). The indirect effect of DII on mortality through eGFR was 0.001 (95% CI: 0.001 to 0.002, *P* < .001), accounting for approximately 9.67% of the total effect. The bootstrap test (1000 resamples) confirmed the significance of the indirect effect, indicating that eGFR partially mediated the association between DII and all-cause mortality.

## 4. Discussion

A defining characteristic of global population dynamics in the 21st century is the continuing intensification of population aging, driven jointly by increasing life expectancy and declining fertility rates. This trend poses multifaceted challenges to public health systems, highlighting the imperative need for a transition towards a healthy aging model.^[26,27]^ In this context, it is crucial to delve into the underlying mechanisms of health risk factors among the elderly population.

This study centered on the pathways linking the DII with all-cause mortality, with particular focus on the potential role of eGFR within this association. eGFR serves as a key indicator of renal function; its decline is a hallmark of CKD, which exhibits a high prevalence among the elderly population. A growing body of research indicates that decreased eGFR is significantly associated with a substantially increased risk of diverse adverse outcomes, including major adverse cardiovascular events and infections.^[28–32]^ Utilizing data from the NHANES (2001–2018), we demonstrated the mediating role of eGFR in the association between the DII and all-cause mortality among individuals aged 60 years and older. The DII is a tool designed to assess the inflammatory potential of an individual’s diet, wherein a higher DII score signifies a diet with greater pro-inflammatory properties.^[33]^ As a modifiable dietary factor, the DII holds significant value for public health interventions. In this large cohort study, we observed a significant positive association between higher DII scores and increased all-cause mortality among older adults (HR = 1.056, 95% CI:1.020–1.094). Furthermore, a dose–response relationship was evident (*P* for trend < .05). Most importantly, mediation analysis revealed that eGFR mediated 9.67% of this association. This suggests that decline in renal function may constitute an important underlying mechanism through which a pro-inflammatory diet elevates mortality risk. Whereas Shivappa et al^[34]^ reported a HR of 1.27 for mortality among individuals in the highest DII tertile using the full-age-range NHANES III sample, their analysis did not conduct age-stratified analyses and did not evaluate the mediating role of renal function. In contrast, the present study specifically focused on individuals aged 60 years and older. After full adjustment for potential confounders, we observed an HR of 1.26, indicating that a pro-inflammatory diet remains an independent predictor of mortality within this specific age group. This finding provides novel epidemiological evidence supporting the persistence of the diet-inflammation-mortality pathway in the elderly population.

The findings of this study demonstrate that elevated DII scores are significantly associated with a constellation of adverse health characteristics, including higher prevalence of obesity, diabetes, and hypertension; suboptimal glycemic and lipid control; reduced eGFR; and ultimately increased all-cause mortality. This pattern suggests that pro-inflammatory dietary patterns may contribute to elevated health risks through multiple pathways. These characteristic associations are closely aligned with existing evidence: Sun et al^[10]^ demonstrated a positive association between elevated DII scores and both all-cause and cardiovascular mortality among U.S. older adults, highlighting that pro-inflammatory diets accelerate organ functional decline through systemic inflammation and metabolic disturbances such as dyslipidemia and insulin resistance. The meta-analysis by Chen et al^[35]^ further confirmed that pro-inflammatory diets significantly increase the risk of liver cancer (relative risk = 2.35), with the mechanism involving chronic liver injury and carcinogenesis driven by inflammatory factors such as IL-6 and C-reactive protein. Notably, the decreased eGFR in the high DII group, along with the metabolic syndrome phenotype characterized by high BMI, elevated UA, and high cholesterol in our study, is consistent with the pathophysiological pathway of “increased disability risk due to low dietary diversity” reported by Kiuchi et al.^[36]^ This suggests that inflammatory diets may contribute to multi-organ dysfunction and increased all-cause mortality by impairing metabolic homeostasis and microvascular function.

Following comprehensive adjustment for confounders, this study confirmed a positive association between the DII and all-cause mortality. When analyzed as a continuous variable, each 1-unit increment in DII was associated with a significant 5.6% increase in mortality risk (HR = 1.056). When categorized into tertiles, only the highest tertile (T3) demonstrated a statistically significant elevation in risk. Critically, our mediation analysis quantified for the first time that eGFR mediates 9.67% of the association between DII and all-cause mortality in older adults, indicating that renal function decline may serve as a key intermediary pathway linking pro-inflammatory diets to mortality risk. These findings demonstrate high concordance with large-scale population studies: Guo et al^[37]^ documented that each 1-unit increase in DII elevated the risk of low eGFR (<60 mL/min/1.73 m^2^) by 16% (OR = 1.16; 95% CI:1.13–1.19) in adults aged ≥ 40 years, revealing a U-shaped dose–response relationship; In a longitudinal cohort of 4554 CKD patients, Yan et al^[38]^ further established that the highest DII tertile had a 21% higher mortality risk (HR = 1.21; 95% CI:1.05–1.39) compared to the lowest tertile, concomitant with significantly reduced eGFR levels. A study in OA identified that high-DII diets accelerated eGFR decline and amplified mortality risk among CKD patients (HR = 1.22), suggesting pro-inflammatory diets may promote renal deterioration through an inflammation-metabolic dysregulation-renal function decline pathway.^[39]^

The underlying pathophysiological mechanism of this association may involve an “inflammatory cumulation-injury-threshold” model. Specifically, chronic exposure to pro-inflammatory diets (high-DII) drives sustained inflammatory responses that progressively induce renal damage and multi-organ functional decline, ultimately elevating all-cause mortality in the population aged ≥ 60 years. Long-term exposure to a high DII diet, rich in processed meats, refined sugars, trans fatty acids, and high sodium but deficient in antioxidant micronutrients such as vitamin C, vitamin E, zinc, and selenium, initially drives a progressive increase in circulating inflammatory markers such as C-reactive protein, IL-6, and TNF-α, establishing a baseline of chronic low-grade inflammation.^[40,41]^ When the persistent inflammatory load gradually exceeds the compensatory threshold of the body’s antioxidant defense and immune clearance, it triggers the “cytokine storm” tipping point. At this stage, the innate immune system is chronically activated, and pro-inflammatory cytokines, together with reactive oxygen species (ROS), act on the renal vascular endothelium and glomerular mesangium. This induces endothelial dysfunction, cellular senescence, and collagen deposition, thereby initiating renal structural remodeling and the fibrosis process.^[42,43]^ Furthermore, the high sugar load associated with a high DII diet can lead to the extensive formation of advanced glycation end-products (AGEs) via the nonenzymatic glycation pathway. After binding with the receptor for AGEs, AGEs rapidly amplify the ROS burst through the nicotinamide adenine dinucleotide phosphate oxidase and nuclear factor-kappa B cascade, resulting in glomerular basement membrane thickening, endothelial dysfunction, and mesangial matrix deposition.^[44]^ Simultaneously, high sodium intake activates the Signal Transducer and Activator of Transcription 1 signaling pathway, inducing the polarization of renal resident macrophages towards the M1 phenotype. These macrophages secrete pro-inflammatory mediators such as IL-6, TNF-α, and C-X-C motif chemokine ligand 1, which exacerbate renal interstitial inflammatory infiltration and fibrosis.^[45]^ AGEs can also induce insulin resistance by downregulating the SIRT1/PGC-1α pathway, maintaining hyperglycemia and forming a vicious cycle of “hyperglycemia-AGEs-kidney” damage.^[44]^ Moreover, the deficiency of antioxidant micronutrients weakens the capacity to clear ROS, exacerbating DNA oxidative damage and cellular senescence, which further injures nephron units.^[46]^ Integrating the aforementioned pathways, a high DII diet synergistically drives the progressive decline in eGFR through a multidimensional interplay involving sustained amplification of inflammatory signals, the AGEs-receptor for AGEs axis, macrophage polarization, and antioxidant-metabolic imbalance.^[37]^ Critically, this sustained eGFR decline constitutes the key mediator linking dietary inflammation to elevated all-cause mortality in the elderly population, thereby validating the tipping point model proposed in the “inflamm-aging” theory wherein chronic inflammation functions as an independent risk factor for renal functional deterioration.^[47]^ Previous studies have indicated that anti-inflammatory dietary patterns, such as the Mediterranean diet, may serve as potential interventions to attenuate renal function decline and reduce mortality.^[48]^ Consequently, anti-inflammatory diets represent a novel protective strategy with potentially transformative clinical implications for advanced-age populations.

The current study further validated the robustness of the association between DII and all-cause mortality across different demographic and metabolic backgrounds through subgroup analyses. Interestingly, despite the consistent overall effect direction, no significant association was observed in the obese subgroup (BMI ≥ 30 kg/m^2^). This finding may align with the “metaflammation” model proposed by Charles-Messance et al, which suggests that obese individuals already exhibit persistent NLRP3 activation and systemic elevation of IL-1β. In this context, the additional pro-inflammatory stimulus from DII may be diluted by an “inflammatory ceiling” effect. Meanwhile, obesity-induced AMP-activated protein kinase/peroxisome proliferator-activated receptor gamma reprogramming can partially counteract dietary inflammatory load through negative feedback loops involving interleukin-10 and transforming growth factor-beta.^[49,50]^ Future studies should employ expanded cohort sizes or incorporate refined obesity phenotyping metrics such as waist circumference and body fat percentage to validate this association.

Based on the nationally representative NHANES large sample, the current study revealed that a pro-inflammatory diet significantly increases the all-cause mortality risk in the elderly population, with 9.67% of this effect mediated by renal function decline. The sufficient follow-up duration and statistical weighting in the study ensured the generalizability of the results. We quantified the overall dietary inflammatory potential using the DII and progressively controlled for confounding factors related to demographics, lifestyle, and chronic diseases through multiple models, thereby enhancing the reliability of causal inferences. Additionally, for the first time, we demonstrated in a general community population that eGFR mediates approximately 9.67% of the effect in the pathway from high DII to all-cause mortality, providing new epidemiological evidence for the diet-kidney-mortality chain.

Although the current study adjusted for multiple confounding factors, unmeasured factors such as physical activity and genetic susceptibility may still lead to residual confounding. Second, the DII score relies on self-reported dietary data, and the absence of regional food components may weaken the precision of inflammatory assessment. Finally, the estimation of eGFR was not combined with cystatin C or urine protein tests, which may limit its sensitivity to early renal damage. Future studies should employ longitudinal dietary repeat measurements and intervention trials to verify causality.

## 5. Conclusion

This study provides empirical evidence supporting a positive association between high DII and all-cause mortality in older adults, while mechanistically establishing the mediating role of eGFR in this relationship. Collectively, these findings strengthen the evidentiary basis for implementing DII monitoring as a strategic approach to early identification of high-risk elderly individuals. Such surveillance enables targeted preservation of renal function through dietary quality improvement, ultimately reducing mortality risk.

## Acknowledgments

We extend our heartfelt appreciation to the editor and reviewers for their meticulous and constructive feedback. Their insights have been instrumental in refining the structure and substance of our research. We are also deeply grateful to the participants and staff of the NHANES for their significant contributions. Our thanks also go to all individuals who have played a part in this endeavor.

## Author contributions

**Conceptualization:** Tingting Hu, Jun Su.

**Data curation:** Ying Zhang.

**Formal analysis:** Tingting Hu, Ying Zhang, Zhu Chen.

**Funding acquisition:** Zhifen Zhang.

**Methodology:** Tingting Hu.

**Resources:** Zhifen Zhang.

**Writing – original draft:** Tingting Hu.

**Writing – review & editing:** Jun Su.
